# Inhalative IL-10 Attenuates Pulmonary Inflammation following Hemorrhagic Shock without Major Alterations of the Systemic Inflammatory Response

**DOI:** 10.1155/2012/512974

**Published:** 2011-10-20

**Authors:** Philipp Kobbe, Philipp Lichte, Helen Schreiber, Lucy Kathleen Reiss, Stefan Uhlig, Hans-Christoph Pape, Roman Pfeifer

**Affiliations:** ^1^Department of Orthopaedic Trauma Surgery, Faculty of Medicine, RWTH Aachen University, Pauwelsstra*β*e 30, 52074 Aachen, Germany; ^2^Institute of Pharmacology and Toxicology, Faculty of Medicine, RWTH Aachen University, Pauwelsstra*β*e 30, 52074 Aachen, Germany

## Abstract

Several studies report immunomodulatory effects of endogenous IL-10 after trauma. The present study investigates the effect of inhalative IL-10 administration on systemic and pulmonary inflammation in hemorrhagic shock. Male C57/BL6 mice (8 animals per group) were subjected to pressure-controlled hemorrhagic shock for 1.5 hrs followed by resuscitation and inhalative administration of either 50 **μ**L PBS (Shock group) or 50 **μ**g/kg recombinant mouse IL-10 dissolved in 50 **μ**L PBS (Shock + IL-10 group). Animals were sacrificed after 4.5 hrs of recovery and serum IL-6, IL-10, KC, and MCP-1 concentrations were measured with ELISA kits. Acute pulmonary inflammation was assessed by pulmonary myeloperoxidase (MPO) activity and pulmonary H&E histopathology. Inhalative IL-10 administration decreased pulmonary inflammation without altering the systemic concentrations of IL-6, IL-10, and KC. Serum MCP-1 levels were significantly reduced following inhalative IL-10 administration. These findings suggest that inhalative IL-10 administration may modulate the pulmonary microenvironment without major alterations of the systemic inflammatory response, thus minimizing the potential susceptibility to infection and sepsis.

## 1. Introduction

Hemorrhagic shock initiates a systemic inflammatory response which is thought to be responsible for the development of ARDS and MOF [1]. The pharmacological modulation of this excessive and uncontrolled release of pro-inflammatory cytokines is considered a promising therapeutic strategy [2]. Previous studies have shown that the administration of IL-10 following hemorrhagic shock is capable of reducing systemic and pulmonary inflammation [3, 4]. However, an early inhibition of the systemic inflammatory response may result in increased rates of infection and sepsis [5–10]. This concern may be diminished by immuno-modulating strategies that target hyperinflammation in specific organs, but leave systemic inflammatory responses unabated. In view of these demands, we hypothesized that inhalative administration of IL-10 following hemorrhagic shock would attenuate the pulmonary but not the systemic inflammatory response.

## 2. Materials and Methods

### 2.1. Animal Care

This research protocol complied with the regulations regarding the care and use of experimental animals published by the NIH and was approved by the Institutional Animal Use and Care Committee of the RWTH Aachen University. Male C57/BL6 mice (Charles Rivers Laboratories, Germany), 6–10 weeks old and weighing 20–30 g, were used in the experiments. The animals were maintained in the Animal Research Center of the RWTH Aachen University with a 12:12 h light-dark cycle and free access to standard laboratory feed and water. Animals were anesthetized with inhaled isoflurane (Abbott Laboratories, Wiesbaden, Germany), 70 mg/kg i.p. pentobarbital (Merial GmbH, Hallbergmoos, Germany), and 0.5 mg/kg buprenorphine (Reckitt-Benckiser, Bergheim, Germany).

### 2.2. Groups 

C57/BL6 mice were divided into four groups. In the Control group (*n* = 8), animals were euthanized after the induction of anaesthesia to obtain physiological baseline levels. Animals in the Sham group (*n* = 8) were subjected to femoral artery catheterization, 1.5 hrs of anaesthesia, and 4.5 hrs of recovery before euthanasia. Animals of the Shock (*n* = 8) or Shock + IL-10 (*n* = 8) group were subjected to femoral artery catheterization, 1.5 hrs of anaesthesia with pressure-controlled hemorrhagic shock followed by inhalative administration of either 50 *μ*L phosphate buffered saline (PBS) or 50 *μ*g/kg recombinant mouse IL-10 (Cys 149 Typ, R&D Systems) dissolved in 50 *μ*L PBS. The animals were sacrificed after 4.5 hrs of recovery.

### 2.3. Femoral Artery Cannulation and Induction of Hemorrhagic Shock

Animals were subjected to anesthesia as described above. A sterile technique was used to perform a left groin exploration, and the left femoral artery was cannulated with tapered polyethylene-10 tubing. The catheter was connected to a digital blood pressure monitor (TSE Systems, Bad Homburg, Germany) and the mean arterial pressure (MAP) was recorded. Pressure-controlled hemorrhagic shock was performed by withdrawing blood over a period of 15 min in a syringe with 0.07 mL of heparin (1000 USP units/mL) until a MAP of 35 mmHg was reached. Hemorrhagic shock was maintained for 1.5 hrs followed by resuscitation with shed blood and an equal volume of 0.9% saline. The catheter was removed, the artery ligated, and the skin incision closed. After a recovery phase of 4.5 hrs the animals were sacrificed.

### 2.4. Administration of Inhalative IL-10

The inhalative administration of PBS or IL-10 was performed using a MicroSprayer Aeroliser (Penn-Century, Philadelphia, Pa, USA) connected to a high-pressure syringe (FMJ-250, Penn-Century, Philadelphia, Pa, USA). The endotracheal intubation was carried out as described by Bivas-Benita et al. [11]. Briefly, the mouth was opened with a blunt forceps and the tongue was pulled out and moved to the left in order to visualise the trachea. The MicroSprayer was inserted into trachea. Once intubation was completed PBS or IL-10 was administered as aerosol. The deposition of aerosols into the small airways using this method has been validated with fluorescent nanoparticles [11].

### 2.5. Blood Collection for Serum Cytokines

Following thoracotomy, cardiac blood was withdrawn under deep anesthesia as part of the procedure of exsanguination for euthanasia. Plasma samples were allowed to clot at 4°C and then were centrifuged at 7000 rpm for 7 min in order to separate the serum from cellular blood components. Serum was stored at −20°C until thawed for further evaluation. Serum IL-6, IL-10, MCP-1, and KC levels were quantified with ELISA kits (R&D System Inc., Minneapolis, Minn, USA) as per manufacturer's specifications. 

### 2.6. Pulmonary Myeloperoxidase (MPO) Activity

To minimize background MPO activity by remaining nonadherent intravascular polymorphonuclear cells, a needle was inserted into the beating right ventricle, after withdrawal of cardiac blood, and the circulation was perfused with 1.5 mL of PBS. The left lung was harvested and immediately snapped-frozen in liquid nitrogen and stored at −80° Celsius. To determine tissue MPO activity, the samples were thawed and homogenized in a lysis buffer according to the manufacturer's protocol. The MPO activity was measured using an MPO-ELISA kit (Hycultec GmbH, Beutelsbach, Germany) and normalized to the protein concentration of the sample (BCA Protein Assay Kit, Pierce, Rockford, Ill, USA).

### 2.7. Pulmonary Histopathology

For the detection of pulmonary inflammation and lung injury the right lung was harvested and immediately fixed in buffered formalin. Paraffin-embedded blocks were cut at 5 *μ*m thickness and stained with H&E (Hematoxylin and Eosin). 

### 2.8. Statistical Analysis 

All results in this paper are expressed as the mean ± SD of eight animals per group. Data were transformed by the BoxCox transformation (JMP 5.0.1 for Windows). In normally distributed variables group comparisons were assessed using ANOVA followed by Tukey's HSD test. Nonnormally distributed parameters were tested using the Kruskal-Wallis-Test. The null hypothesis was rejected for *P* < 0.05. Data were analysed using SPSS Version 18 (SPSS, Chicago, Ill, USA).

## 3. Results

### 3.1. Serum Cytokine Levels

**Figure 1:**
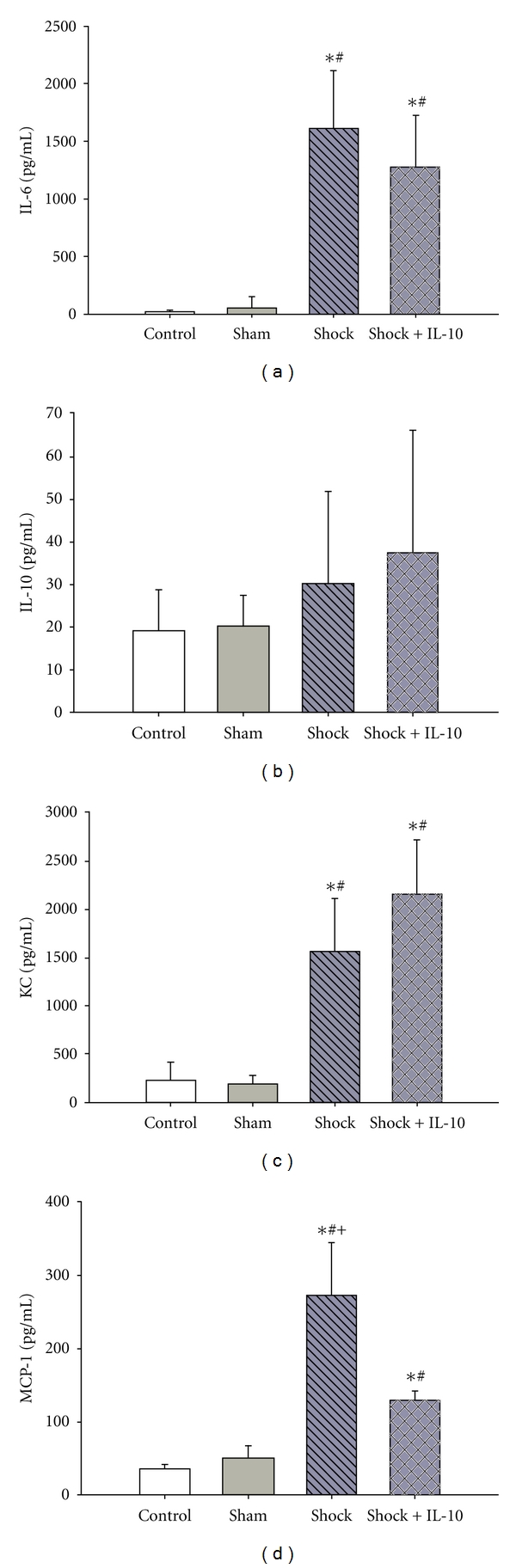
Comparison of serum IL-6 (a), IL-10 (b), KC (c), and MCP-1 (d) levels in C57/BL6 mice following hemorrhagic shock with (Shock + IL-10) or without (Shock) inhalative administration of IL-10. Results are expressed as means ± SD of 8 animals per group (*P < 0.05 versus Control; ^#^ *P* < 0.05 versus Sham; ^+^ *P* < 0.05 versus Shock + IL-10).

Serum IL-6 levels were significantly higher in animals subjected to hemorrhagic shock as compared to Control and Sham animals. However, the serum IL-6 concentration was indistinguishable between Shock and Shock + IL-10 animals, thus inhalative IL-10 did not significantly alter systemic IL-6 levels (Figure 1(a)). 

Serum IL-10 concentrations showed an increase following hemorrhagic shock; however this did not reach statistical significance. Inhalative IL-10 was not associated with a significant increase in systemic IL-10 levels (Figure 1(b)).

Serum KC levels were significantly increased in Shock and Shock + IL-10 animals as compared with Control and Sham mice. There was no significant difference in serum KC levels between Shock and Shock + IL-10 mice (Figure 1(c)).

Serum MCP-1 levels were significantly higher following hemorrhagic shock as compared to Control and Sham animals. Furthermore, the MCP-1 concentration was significantly reduced in animals with inhalative IL-10 administration, thus inhalative IL-10 alters systemic MCP-1 levels (Figure 1(d)).

### 3.2. Pulmonary Inflammation

**Figure 2:**
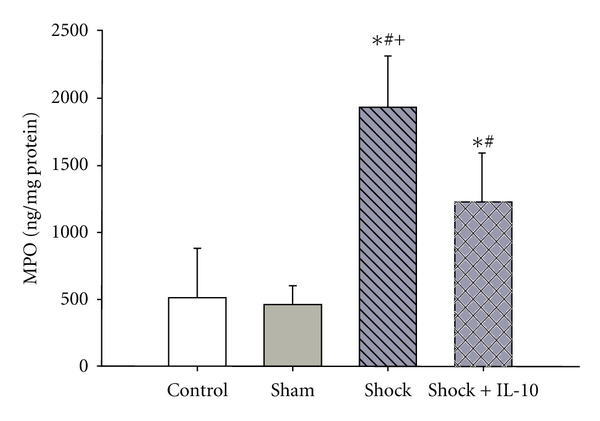
Pulmonary myeloperoxidase (MPO) activity in C57/BL6 mice following hemorrhagic shock with (Shock + IL-10) or without (Shock) inhalative administration of IL-10. Results are expressed as means ± SD of 8 animals per group (*P < 0.05 versus Control; ^#^ *P* < 0.05 versus Sham; ^+^ *P* < 0.05 versus Shock + IL-10).

Pulmonary MPO activity was significantly higher in the Shock and Shock + IL-10 group as compared with Control and Sham animals. Inhalative IL-10 significantly reduced the pulmonary MPO activity as compared to Shock animals (Figure 2).

**Figure 3:**
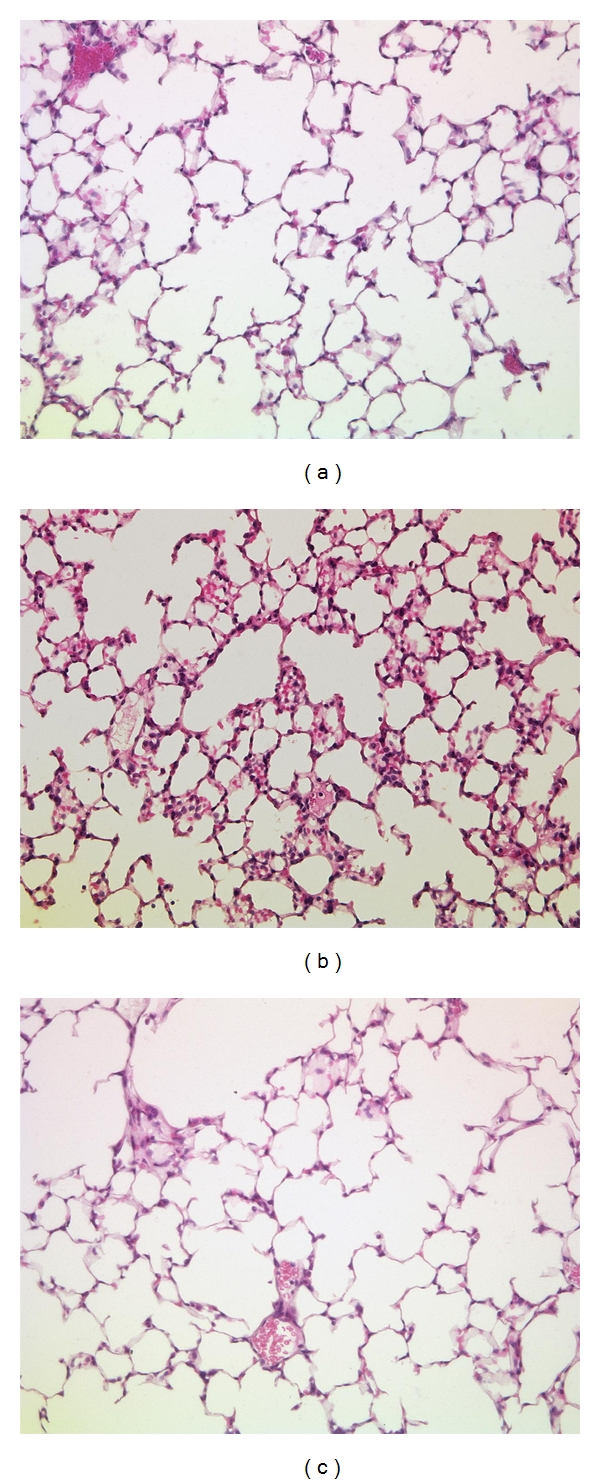
Representative H&E (Hematoxylin and Eosin) lung histology (20x) of the Control (a), Shock (b), and Shock + IL-10 (c) group 4.5 hrs after resuscitation. Inhalative IL-10 reduces the pulmonary influx of inflammatory cells.

This effect was confirmed by histology which showed a reduced pulmonary infiltration with inflammatory cells (Figure 3).

## 4. Discussion

The immunoregulatory potential of IL-10 is well recognized and a potential role of IL-10 as a therapeutic agent is increasingly investigated in various animal models [3, 4, 12–14] and human studies [12]. Beneficial effects of early systemic IL-10 release following injury have been reported [3, 15], mainly related to the inhibition of proinflammatory cytokine synthesis [16, 17] and leukocyte recruitment [18]. However, especially in the later course following trauma, the incidence of infection is strongly associated with systemic IL-10 concentrations [5–10]. Thus, systemic IL-10 is a double-edged sword in treating severe trauma: potentially beneficial in the early but deleterious in the later phase. This conundrum raises the possibility whether locally applied IL-10 strategy could possibly protect end organs by changing their inflammatory microenvironment without altering the systemic inflammatory response and the susceptibility to infection and sepsis. The lung is frequently affected by systemic inflammation and the development of acute lung injury seems to be associated with local and systemic IL-10 concentrations, examples are hemorrhage [3, 4], peritonitis [19], or major surgery [20]. Low IL-10 concentrations in the BAL of patients already suffering from ARDS were correlated with an increased mortality rate [21], whereas patients having an IL-10 polymorphism with increased IL-10 release showed a decreased incidence of ARDS [22]. Given that hemorrhagic shock impairs the induction of IL-10 expression by alveolar macrophages [23] and that systemic IL-10 administration attenuates pulmonary but not hepatic injury [4, 13], we hypothesized that a local repletion of pulmonary IL-10 following hemorrhagic reduces pulmonary inflammation without altering the systemic inflammatory response. 

Trauma hemorrhage is well known to lead to pulmonary inflammation and leukocyte infiltration, that is at least in part mediated by KC [24]. In the present study, we observed significant increases of systemic IL-6 and KC concentrations following hemorrhagic shock and both effects were not altered by inhalative IL-10 administration. These findings are in contrast to studies reporting significantly reduced systemic IL-6 and KC levels in hemorrhagic shock following intra-arterial, intraperitoneal, or subcutaneous IL-10 administration [3, 13, 25]. These findings support our hypothesis that inhalative administered IL-10 mainly affects the lung without major alterations of the systemic inflammatory response. This is in accordance with no detectable differences of systemic IL-10 concentrations between animals subjected to hemorrhagic shock with or without inhalative IL-10, although we cannot exclude an earlier systemic IL-10 peak, due to the short half-life of IL-10.

Interestingly, we observed a significant reduction of systemic MCP-1 following inhalative IL-10 application. MCP-1 is a major attractant for macrophages and monocytes and is upregulated following trauma hemorrhage [26]. An explanation for reduced systemic MCP-1 concentrations may lie in the role of alveolar macrophages which secrete MCP-1 and thereby potentiate inflammatory triggered lung injury [27, 28]. Thus systemic measured MCP-1 levels following hemorrhagic shock may be derived from the secretion of alveolar macrophages.

We have previously observed these specific modulations of chemokine release in a model of IL-10 KO mice [4]. Herein, IL-10 deficiency significantly increased systemic MCP-1 but not KC concentrations. Yet, we are not able to explain this selective modulation of chemokines by IL-10, but it may be speculated that, in contrast to the systemic MCP-1 concentration, the alveolar macrophages are not the main source for systemic measured KC levels. 

The influx of inflammatory cells in the lung following hemorrhagic shock has been well described and is thought to be a major contributor to the development of remote organ dysfunction following trauma [29, 30]. Our data show that inhalation of IL-10 significantly reduces pulmonary myeloperoxidase activity, an established marker for pulmonary neutrophil infiltration, in shocked animals. Pulmonary histopathology showed a trend towards a decreased influx of inflammatory cells; however signs of severe pulmonary injury were not detectable in this early phase. This is in line with other studies detecting histological evidence of ARDS usually not before 24 hrs after hemorrhagic shock [4]. Overall, the reduced pulmonary inflammation is in accordance with other studies showing that IL-10 administration significantly decreased neutrophil infiltration in the lung [3, 31, 32]. The beneficial pulmonary effect of endogenous IL-10 has also been shown in other animal models [13, 33] and is further supported by clinical studies which report that nonsurvivors of ARDS had significantly lower levels of IL-10 in the BAL-fluid as compared to survivors [34]. 

Several features and limitations of our study merit further comment. We did not investigate later time points and one may criticize that inhalative IL-10 administration may delay but not prevent pulmonary inflammation following hemorrhagic shock. Further, the unchanged systemic concentrations of IL-6, KC, and IL-10 do not rule out that other important mediators in the posttraumatic inflammatory cascade may be altered by inhalative IL-10 and thereby the susceptibility to infection may again be increased.

In conclusion, this study demonstrates that inhalative IL-10 reduces pulmonary inflammation following hemorrhagic shock without major alterations in the systemic inflammatory response. This may be a valuable therapeutic strategy because inhalative IL-10 administration may solve the dilemma of the two-edged sword: end organ protection is possible without altering the systemic inflammatory response and the susceptibility to infection and sepsis. Nonetheless, further studies are required to elucidate the complex immunomodulatory action of IL-10 under the condition of hemorrhagic shock.

## Abbreviations

ARDS: Adult Respiratory Distress Syndrome
IL-6: Interleukin-6
IL-10: Interleukin-10
KC: Keratinocyte-derived Cytokine
MCP-1: Macrophage/Monocyte Chemotactic Protein-1
MOF: Multiple Organ Failure
MPO: Myeloperoxidase
PBS: Phosphate Buffered Saline.
